# Whole-Genome Sequencing for Drug Resistance Profile Prediction in *Mycobacterium tuberculosis*

**DOI:** 10.1128/AAC.02175-18

**Published:** 2019-03-27

**Authors:** Sebastian M. Gygli, Peter M. Keller, Marie Ballif, Nicolas Blöchliger, Rico Hömke, Miriam Reinhard, Chloé Loiseau, Claudia Ritter, Peter Sander, Sonia Borrell, Jimena Collantes Loo, Anchalee Avihingsanon, Joachim Gnokoro, Marcel Yotebieng, Matthias Egger, Sebastien Gagneux, Erik C. Böttger

**Affiliations:** a Swiss Tropical and Public Health Institute, Basel, Switzerland; b University of Basel, Basel, Switzerland; c Institute of Medical Microbiology, University of Zürich, Zürich, Switzerland; d National Center for Mycobacteria, University of Zürich, Zürich, Switzerland; e Institute of Social and Preventive Medicine, University of Bern, Bern, Switzerland; f HIV-NAT/Thai Red Cross AIDS Research Centre, Bangkok, Thailand; g TB Research Unit, Department of Medicine, Faculty of Medicine, Chulalongkorn University, Bangkok, Thailand; h Centre de Prise en Charge de Recherche et de Formation, Yopougon, Abidjan, Côte d'Ivoire; i College of Public Health, Ohio State University, Columbus, Ohio, USA; j Centre for Infectious Disease Epidemiology and Research, University of Cape Town, Cape Town, South Africa; k Instituto de Medicina Tropical Alexander von Humboldt, Universidad Peruana Cayetano Heredia, Lima, Peru; l Institute for Infectious Diseases, University of Bern, Bern, Switzerland

**Keywords:** drug resistance, drug resistance level prediction, *Mycobacterium tuberculosis*, quantitative phenotypic drug susceptibility testing, whole-genome sequencing

## Abstract

Whole-genome sequencing allows rapid detection of drug-resistant Mycobacterium tuberculosis isolates. However, the availability of high-quality data linking quantitative phenotypic drug susceptibility testing (DST) and genomic data have thus far been limited. We determined drug resistance profiles of 176 genetically diverse clinical M. tuberculosis isolates from the Democratic Republic of the Congo, Ivory Coast, Peru, Thailand, and Switzerland by quantitative phenotypic DST for 11 antituberculous drugs using the BD Bactec MGIT 960 system and 7H10 agar dilution to generate a cross-validated phenotypic DST readout. We compared DST results with predicted drug resistance profiles inferred by whole-genome sequencing. Classification of strains by the two phenotypic DST methods into resistotype/wild-type populations was concordant in 73 to 99% of cases, depending on the drug. Our data suggest that the established critical concentration (5 mg/liter) for ethambutol resistance (MGIT 960 system) is too high and misclassifies strains as susceptible, unlike 7H10 agar dilution. Increased minimal inhibitory concentrations were explained by mutations identified by whole-genome sequencing. Using whole-genome sequences, we were able to predict quantitative drug resistance levels for the majority of drug resistance mutations. Predicting quantitative levels of drug resistance by whole-genome sequencing was partially limited due to incompletely understood drug resistance mechanisms. The overall sensitivity and specificity of whole-genome-based DST were 86.8% and 94.5%, respectively. Despite some limitations, whole-genome sequencing has the potential to infer resistance profiles without the need for time-consuming phenotypic methods.

## Abstract (precis)

Whole-genome sequencing allows rapid detection of drug-resistant Mycobacterium tuberculosis isolates. However, the availability of high-quality data linking quantitative phenotypic drug susceptibility testing (DST) and genomic data have thus far been limited.

## INTRODUCTION

Timely and accurate drug susceptibility testing (DST) of M. tuberculosis isolates is vital to prevent the transmission of multidrug-resistant strains (MDR; resistance to rifampin and isoniazid) (1). The slow growth and stringent biosafety requirements of M. tuberculosis make obtaining a full DST profile by culture-based techniques a matter of weeks or months. In addition, culture-based DST is notoriously challenging for several drugs, e.g., pyrazinamide and ethionamide, due to poor drug solubility in commonly used culture media (2).

Drug resistance in M. tuberculosis is mainly conferred by chromosomal mutations in a few genes (3), making it possible to detect drug resistance by sequencing these genes or probing them by molecular hybridization (4). Several commercial tests for the detection of resistance-associated mutations are available, e.g., the GenoType MTBDRplus V2 (Hain Lifescience GmbH, Nehren, Germany) (5) and the AID TB Resistance line probe assay (AID GmbH, Strassberg, Germany) (6). The World Health Organization (WHO) endorses line probe assays and the Xpert MTB/RIF assay (Cepheid, Sunnyvale, CA, USA) for the detection of rifampin resistance as a surrogate marker for multidrug resistance (7, 8). These molecular tests have high sensitivities for drugs with an established target(s) of resistance and for which only a few mutations are responsible for most resistance *in clinico* (e.g., rifampin and isoniazid) (4). However, these molecular tests show low sensitivity for heteroresistant strains (concomitant presence of the wild type [wt] and mutant or multiple different resistance variants in patient isolates) when frequencies of mutant variants drop below 5 to 50% (9, 10). Furthermore, there are no commercially available rapid tests for many currently used or prospective drugs (e.g., bedaquiline, delamanid, linezolid, and p-aminosalicylic acid), and the WHO only recently defined *ad interim* critical concentrations for bedaquiline and delamanid for use with the Bactec MGIT 960 system (11, 12).

A wealth of genomic data on drug-resistant M. tuberculosis has become available in recent years (13, 14). Unfortunately, quantitative phenotypic DST data are lacking for most of the genetic data sets, which are necessary to infer phenotypes from genotypes. In addition, DST data are often limited, as the strains were classified as susceptible or resistant using the WHO-defined critical concentration (15). There is an urgent need to link genotypic and phenotypic drug resistance readouts to obtain a better understanding of the mechanisms influencing the evolution and spread of drug resistance in M. tuberculosis (3, 16).

Whole-genome sequencing (WGS) of clinical isolates allows for accurate identification of established chromosomal mutations increasing the MIC (13, 17, 18) and may ensure adequate treatment in days instead of months. We compared whole-genome-based drug resistance profiles with two culture-based quantitative DST methods for a total of 11 drugs, including rifampin, rifabutin, isoniazid, all WHO group B drugs (streptomycin, kanamycin A, amikacin, and capreomycin), and selected group A (moxifloxacin), group C (ethionamide), and group D (ethambutol and pyrazinamide) drugs (11).

## RESULTS

### Agreement between MGIT 960 and 7H10 agar dilution phenotypic DST.

**FIG 1:**
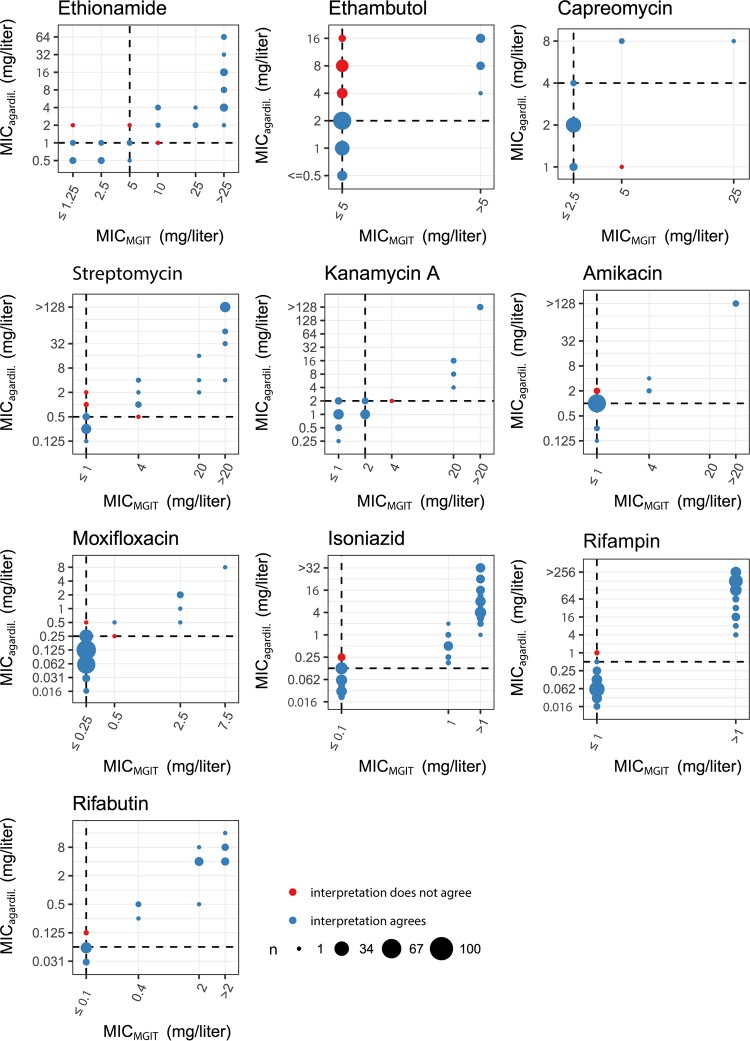
Method agreement between phenotypic DST performed with MGIT 960 and 7H10 agar dilution (agardil.), represented as Bland-Altman plots for all drugs tested in this study.

Table 1 and Fig. 1 summarize the agreement between the semiquantitative/quantitative MIC determination by MGIT 960 and 7H10 agar dilution in terms of classifying strains as belonging to the resistotype or wt populations, as inferred by growth/no growth at the epidemiological cutoffs (ECOFF) (Table 2). Agreement was high for all drugs except ethambutol (see below). For most drugs, the MGIT 960-based MICs were higher than the 7H10 agar dilution-based MICs. MICs obtained using the two methods were within 1 to 2 2-fold dilution steps of each other. The classifications into resistotype/wt populations demonstrated high rank correlations for most drugs (Table 1 and Fig. 1), except for capreomycin (see Fig. S4 in the supplemental material), due to few strains demonstrating increased capreomycin MICs included in the study.

**TABLE 1:** Summary statistics of the method agreement between 7H10 agar dilution and MGIT 960-based phenotypic DST for all drugs assayed in this study

Antibiotic	*n*	Categorical agreement (%)	SD of log_2_(MIC MGIT 960/MIC agar dilution)	γ
Ethionamide	56	95	1.9 ± 0.3	0.91
Ethambutol	171	73	1.9 ± 0.5	0.94
Capreomycin	56	98	1.5 ± 0.5	0.65
Streptomycin	56	93	1.5 ± 0.3	0.98
Kanamycin A	56	98	1.2 ± 0.2	0.8
Amikacin	174	98	1.4 ± 0.6	1
Moxifloxacin	173	99	1 ± 0.2	1
Isoniazid	173	96	1.2 ± 0.1	1
Rifampin	174	99	NA	1
Rifabutin	56	96	0.8 ± 0.1	0.98

**TABLE 2:** ECOFF used for 7H10 agar dilution and MGIT 960 phenotypic DST, derived from wt MIC distributions determined in this study

Antibiotic	ECOFF^*a*^ (mg/liter)	
	Agar dilution	MGIT 960
Ethionamide	1 (5)	5
Ethambutol	2 (5)	5
Capreomycin	4	2.5
Streptomycin	0.5 (2)	1
Kanamycin A	2 (5)	2 (2.5)
Amikacin	1 (4)	1
Moxifloxacin	0.25 (0.5)	0.25 (0.5)
Isoniazid	0.125 (0.2)	0.1
Rifampin	0.5 (1)	1
Rifabutin	0.0625	0.1
Pyrazinamide	NA	100

a The values given in parentheses are the critical concentrations recommended by the WHO in 2014 (43). NA, not applicable.

### WGS and *in silico* resistance profile prediction.

**FIG 2:**
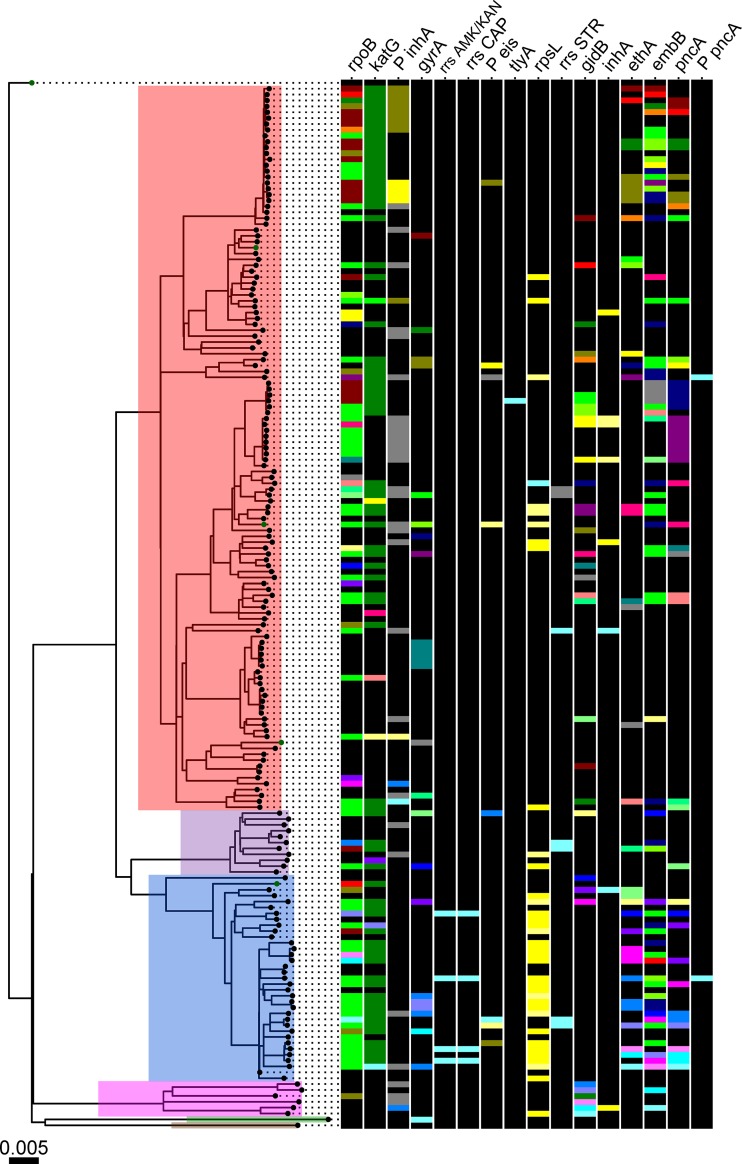
Maximum likelihood phylogeny of 176 M. tuberculosis strains based on 20,510 variable positions. Reference strains are labeled with green. Main lineages are highlighted with the following color scheme: red, L4; purple, L3; blue, L2; pink, L1; green, L6; brown, L5. Scale bar indicates the number of substitutions per site. Phylogeny is rooted on M. canettii. Colored bars indicate resistance mutations per gene, and within a distinct column (gene) each colored bar represents a distinct mutation. Black bars indicate no mutation, i.e., wt.

A total of 176 whole-genome sequences with a median coverage of 67.6× (interquartile range [IQR], 37.48) were obtained. Median mapping percentage and percentage of genome covered were 98.7% (IQR, 0.94) and 99.4% (IQR, 0.4), respectively. Genes involved in drug resistance demonstrated high coverages, with only 0.8% of all positions suffering from coverages below 7× (see the supplemental material). All major M. tuberculosis lineages, except lineage 7, were represented in the study (L1 = 6, L2 = 36, L3 = 11, L4 = 123, L5 = 1, and L6 = 1). The strains showed a range of drug resistance profiles (Fig. 2). Based on the set of analyzed genes (Table 3), 25 strains were predicted to be fully susceptible against all assayed drugs, 59 strains were mono-/polyresistant, 91 strains were MDR, and two strains were predicted to be extensively drug resistant (XDR; isoniazid, rifampin, fluoroquinolone, and aminoglycoside resistant).

**TABLE 3:** List of genes implicated in drug resistance in M. tuberculosis that were screened for polymorphisms by WGS^*a*^

Drug	Target gene(s)
Ethionamide	*ethA, inhA, inhA* promoter
Ethambutol	*embB*
Capreomycin	*rrs, eis* promoter, *tlyA*
Streptomycin	*rrs, gidB, rpsL*
Kanamycin A	*rrs, eis* promoter
Amikacin	*rrs, eis* promoter
Moxifloxacin	*gyrA*
Isoniazid	*katG, inhA* promoter
Rifampin/rifabutin	*rpoB*
Pyrazinamide	*pncA, pncA* promoter

a Data are adapted from references 3, 12, and 23.

### Drug resistance profile prediction by WGS versus phenotypic DST.

After exclusion of known phylogenetic markers not involved in resistance, WGS-based resistotype prediction using a defined set of target genes (Table 3) was highly congruent with the categorical classification based on the phenotypic DST for most drugs (Tables 1 and 4 and Fig. 1). Based on the *in silico* resistotype prediction, the MICs of mutant and wt strains frequently followed a Gaussian distribution. However, the same resistance marker may confer different MICs in different strains (Fig. S1C, S2C, S3C, S8C, S9C, and S10C). In some cases, the increase in the MIC conferred by a certain resistance mutation fell within the distribution of the wt MIC (e.g., for *gidB* and *eis* promoter mutations; Fig. S3C and S6C).

### Distinct wt and mutant MIC distributions.

MIC distributions of wt and mutant strains were well separated for rifampin, rifabutin, isoniazid, kanamycin A, amikacin, capreomycin, streptomycin, and pyrazinamide, indicating that the resistance markers used had a high positive predictive power (88.9% overall positive predictive power of association with MIC increases). For streptomycin, two strains harbored no mutations in the target genes, yet they demonstrated high-level phenotypic resistance (Fig. S3C).

### Overlapping wt and mutant MIC distributions.

**FIG 3:**
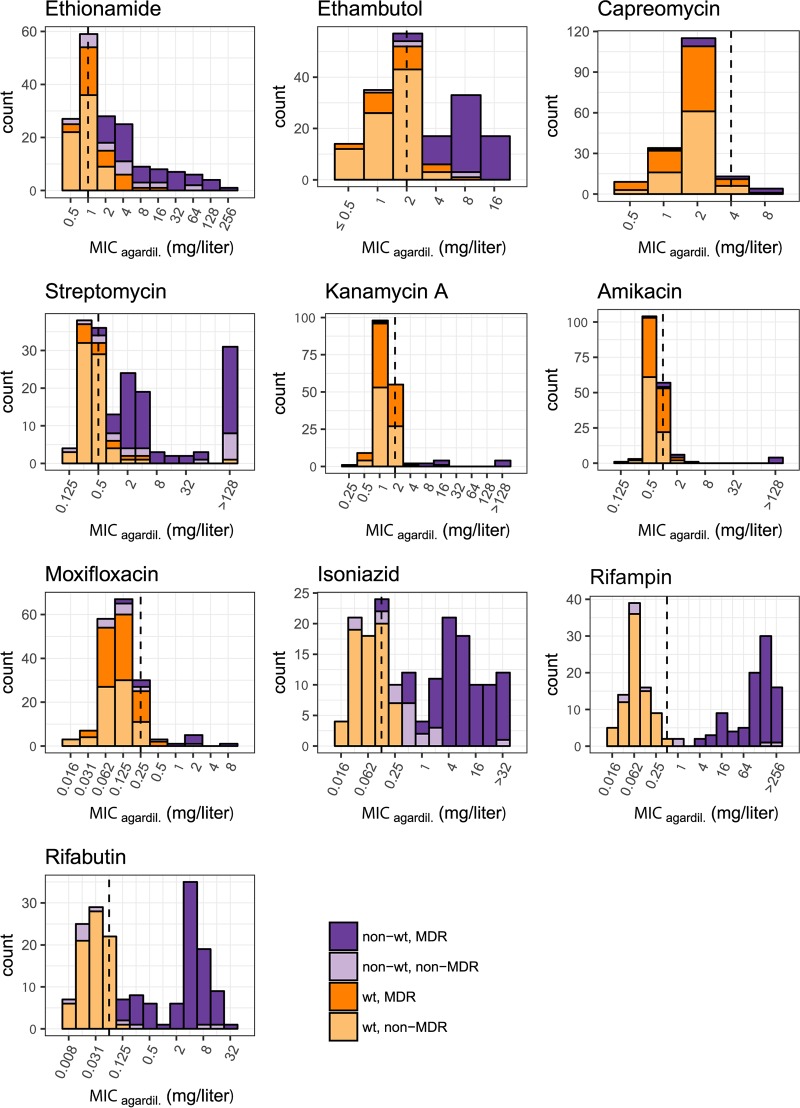
Histograms of MICs (7H10 agar dilution) for all drugs assayed in this study.

MIC distributions of wt and mutant strains overlapped for ethambutol, moxifloxacin, and ethionamide (Fig. 3). For ethambutol and ethionamide, overlapping MIC distributions of wt and mutant strains were associated with a large number of polymorphisms in resistance-conferring genes (ethambutol resistance, 22 polymorphisms in *embB*; ethionamide resistance, 28 in *ethA*, 3 in *inhA*, and 6 in the *inhA* promoter). Solubility issues with ethionamide led to quantitative differences in MGIT 960 versus 7H10 agar dilution-based DST (Table 1 and Fig. 1). The overlap in MIC distributions between the wt and strains carrying an *embB* mutation was reduced by adjusting the critical concentration for ethambutol resistance from 5 mg/liter to 2.5 mg/liter (MGIT 960). However, there was variability in the MICs for the same mutation (e.g., MIC EmbB M306I/V in 7H10 agar dilution, 4 to 16 mg/liter; Fig. S2C). Moxifloxacin resistance was rare (*n* = 9, MGIT 960, critical concentration of 0.25 mg/liter), and MIC distributions of mutant strains partially overlapped those of the wt. Sensitivity of the genome-based moxifloxacin resistance prediction was 80.0% (Table 4).

**TABLE 4:** Sensitivity and specificity of the genome-based drug resistance profile prediction^*a*^

Drug	Sensitivity (%)	Specificity (%)
Ethionamide	75.0	92.9
Ethambutol	89.6	94.2
Capreomycin	75.0	94
Streptomycin	68.0	92.1
Kanamycin A	83.3	98.8
Amikacin	63.6	96.9
Moxifloxacin	80.0	90.2
Isoniazid	93.6	96.8
Rifampin	100	94.0
Rifabutin	98.9	94.0
Pyrazinamide	80.8	88.9

a Sensitivity and specificity were determined using the 7H10 agar dilution-based categorical classification as the gold standard for all drugs except pyrazinamide, for which the MGIT 960 categorical classification was used.

### Defining cutoffs for high- and low-level MICs.

**(i) Isoniazid.** Mutations in the promoter of *inhA* caused low-level MICs of <1 mg/liter (7H10 agar dilution) compared to strains harboring mutations in *katG* or combinations of *inhA* promoter and *katG* mutations, which demonstrated MIC levels ranging from ≥1 mg/liter to >32 mg/liter in 7H10 agar dilution (Fig. S8C). Defining cutoffs for low-level (≤1 mg/liter for MGIT 960/7H10 agar dilution) and high-level (>1 mg/liter MGIT 960/7H10 agar dilution) isoniazid MICs is warranted.

**(ii) Rifampin/rifabutin.** Most mutations in *rpoB* increased the MIC for rifamycins beyond the therapeutic window (peak serum concentration, 10 mg/liter [19, 20]). However, some rare *rpoB* mutations (e.g., RpoB L452P, H445L; Fig. S9C) demonstrated MICs within the therapeutic window. Thus, defining cutoffs for low- and high-level MICs may be justified.

For rifampin, cutoffs for low- and high-level MICs were ≤4 and 2 mg/liter for MGIT 960/7H10 agar dilution and >4 and 2 mg/liter for MGIT 960/7H10 agar dilution.

For rifabutin, our data suggest a cutoff for low- and high-level MICs of ≤0.4 and 0.25 or 0.5 mg/liter for MGIT 960/7H10 agar dilution and >0.4 and 0.25 or 0.5 mg/liter for MGIT 960/7H10 agar dilution.

**FIG 4:**
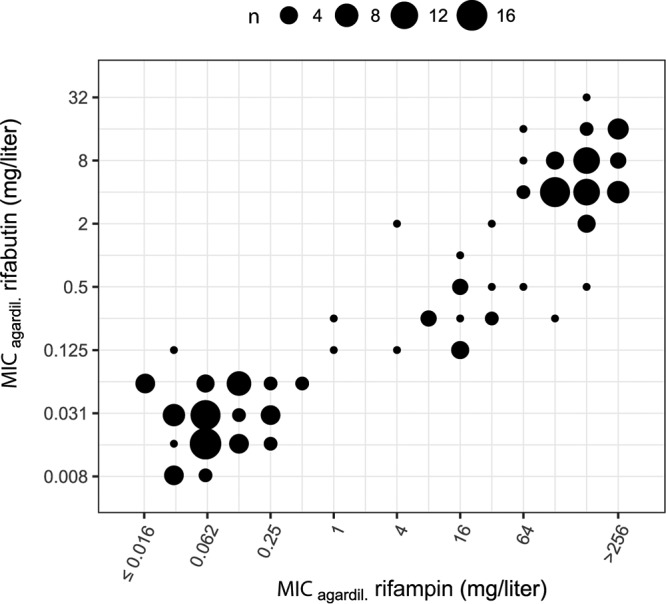
Correlation between 7H10 agar dilution MICs for rifampin and rifabutin.

Mutations in *rpoB* conferring resistance to rifampin and rifabutin showed highly correlated increases (Fig. 4) of MICs beyond the therapeutic window for most *rpoB* mutations (Fig. 3 and Fig. S9C and S10C), indicating that both drugs are rendered clinically ineffective by the mutations identified in the data set (21) and cannot substitute for each other.

**(iii) Amikacin.** Few strains had mutations in the regions of *rrs* relevant for amikacin resistance or the *eis* promoter (*n* = 12). Mutations in *rrs* were associated with high-level MICs (>128 mg/liter in 7H10 agar dilution). With regard to the *eis* promoter, only the C-14T mutation increased the MIC and led to low-level MICs (2 to 4 mg/liter in 7H10 agar dilution). The definition of a cutoff for low-level (≤4 mg/liter for MGIT 960/7H10 agar dilution) and high-level (4 mg/liter for MGIT 960/7H10 agar dilution) amikacin MICs may be warranted.

**(iv) Streptomycin.** Certain mutations lead to MICs well beyond the therapeutic window (19) of streptomycin (e.g., RpsL K43R, MIC 7H10 agar dilution of >128 mg/liter; Fig. S3C). On the other hand, *gidB* mutations increase the MIC only moderately (MIC 7H10 agar dilution, 1 to 4 mg/liter; Fig. S3C). Mutational combinations in *gidB*, *rrs*, and *rpsL* were common and produced a range of different MICs. Despite the distribution of MICs conferred by combinations of mutations, there were distinct mutations that systematically led to MICs beyond the therapeutic window, e.g., RpsL K43R. Defining a cutoff for low-level (MGIT 960, ≤4 mg/liter; 7H10 agar dilution, ≤4 to 8 mg/liter) and high-level streptomycin MICs (MGIT 960, >4 mg/liter; 7H10 agar dilution, >4 to 8 mg/liter) is warranted.

## DISCUSSION

The results of MGIT 960 and 7H10 agar dilution-based phenotypic DST methods were highly correlated and suitable to separate the resistotype from the wt populations. Based on phenotypic DST results and WGS, we were able to define cutoffs for high- and low-level MICs for isoniazid, rifampin, streptomycin, and amikacin. Defining such cutoffs may serve as starting points for correlating mutational, DST, and pharmacokinetic/dynamic data to gain more insight into the influence of individual mutations on treatment outcomes, especially in the light of, e.g., increased drug dosing.

Our data suggest that the current WHO-defined critical concentration for phenotypic DST of ethambutol by MGIT 960 (5 mg/liter) is too high and may misclassify strains as belonging to the wt population compared to the 7H10 agar dilution-based classification. Given the narrow therapeutic window for ethambutol, this may lead to mistreatment due to presumed ethambutol susceptibility. After adjusting the ECOFF to 2.5 mg/liter for MGIT 960, we observed a strong improvement of the categorical agreement between MGIT 960- and 7H10 agar dilution-based classification into resistotype/wt populations.

The mutations identified by WGS had a high predictive power to classify strains as belonging to the resistotype population. However, the predictive power depends on a number of factors. For instance, the increase in MIC conferred by an identical mutation can vary greatly in different strains (e.g., EmbB M306I/V and RpsL K88R) (22). Such variation may be clinically relevant if there is a significant overlap between the MICs of mutant and wt strains (23), as was the case for strains harboring mutations in genes associated with ethionamide, ethambutol, and streptomycin (e.g., *gidB*) resistance. Furthermore, it is difficult to classify strains as part of resistotype or wt populations if the MIC increase lies within the therapeutic window of a drug. The overlap between MICs of mutant and wt strains is confounded by the fact that we only screened for mutations in genes which had previously been associated with drug resistance. Thus, we might have missed possible resistance-conferring mutations in other genes. Additionally, WGS will always produce distributions of coverages, which in turn will inevitably lead to certain regions in the genome suffering from low coverage, preventing the detection of mutations. However, in cases where we observed elevated MICs without any mutations detected in the target genes, coverage issues could not explain the absence of any mutations. Furthermore, the strain genetic background (24), nonmutational mechanisms (e.g., modulation of gene expression) (25), and drug efflux mechanisms (26) may contribute to the variability in increase of the MIC conferred by resistance mutations.

The predictive power of mutations in target genes also depends on removing phylogenetic markers not involved in increasing MICs. Separating phylogenetic from resistance-associated markers works well for essential (highly conserved) genes such as *rpoB*, *rpsL*, and *rrs* but is problematic in nonessential genes involved in the conversion of prodrugs into their active forms, like *pncA* (pyrazinamide) and *ethA* (ethionamide), or in genes that generally exhibit higher numbers of polymorphisms, e.g., *embB*. Of note, the *embABC* operon is highly polymorphic, harboring more polymorphisms than expected by chance (mutations in *embABC* operon, 81; expected, 44.8; *P* = 9.174e−07, binomial test). Mutations conferring increased ethambutol MICs (27) will therefore inevitably evolve in the presence of phylogenetic single-nucleotide polymorphisms (SNPs) and may interact epistatically to produce the variability in MICs we observed for wt strains and for the most common marker associated with increased ethambutol resistance MICs, *embB* M306I/V. The *embABC* operon is involved in the biosynthesis of decaprenylphosphoryl-β-d-arabinose, which is an integral component of the mycobacterial cell wall. The cell envelope interacts with the host immune system, and the high levels of diversity of these genes might be the product of diversifying selection due to host immune pressure. The influence of polymorphisms in the *embABC* operon on MICs in general is supported by the observation that subinhibitory concentrations of ethambutol lower the MICs for isoniazid, rifampin, and streptomycin (28). Thus, even small changes in activity of the decaprenylphosphoryl-β-d-arabinose biosynthetic and utilization pathway might alter cell wall permeability and influence MICs of several drugs.

Similarly, in the case of increased streptomycin MICs, the RpsL substitution K88R exhibited a wide range of MIC increases, partially within the therapeutic window of the drug. Streptomycin was the first effective antituberculous drug discovered (29) and has been used for decades. The long-term use has produced complex resistance profiles with multiple mutations known to increase streptomycin MICs on their own (e.g., in *gidB*, *rpsL,* and *rrs*) occurring concomitantly, producing wide ranges of MICs. Furthermore, many strains with increased streptomycin MICs displayed MDR/XDR phenotypes. Mutations conferring increased streptomycin MICs are frequently found in backgrounds which have mutations in genes affecting the information pathway (DNA→RNA→proteins), e.g., *gyrA* (DNA gyrase), *rpoB* (DNA-dependent RNA polymerase), and *rrs* (rRNA). The simultaneous presence of multiple MIC-increasing mutations may alter the adaptive landscape (30, 31). In addition, nonmutational processes (e.g., alteration of gene expression) may compensate for fitness costs of drug resistance and at the same time alter the MIC for the drug (25). This has not been demonstrated for streptomycin resistance in M. tuberculosis, but it seems possible that compensation of fitness costs in MDR phenotypes alter the MIC for streptomycin (30), considering that streptomycin is not part of the current standard treatment regimen and selection for high-level streptomycin MICs is relaxed.

Concerning *eis* promoter mutations and aminoglycoside resistance, there is mounting evidence that the *eis* C-14T promoter mutation confers clinically relevant increases in amikacin MICs, especially in the light of the revised critical concentrations for amikacin (2 mg/liter for 7H10 agar dilution) (11).

We observed an overrepresentation of lineage 2 and 4 strains in our sample set. The strain set used to establish the methodology was collated with a specific aim to include drug-resistant strains. Given the frequent association of lineages 2 and 4 with drug resistance (32, 33), the observed skew is not surprising. Furthermore, lineage 2 and 4 strains are also frequently isolated at the collection sites of the strain set used to apply the methodology (Ivory Coast, Peru, and Democratic Republic of the Congo). Similarly, increased MICs for a number of drugs (amikacin, capreomycin, kanamycin, and moxifloxacin) were rare, reflecting the scarcity of pre-XDR/XDR phenotypes in Switzerland and at the sites of prospective sampling.

At 63.6% to 80.8%, sensitivities were low (34) for a number of drugs (i.e., amikacin, moxifloxacin, and pyrazinamide) (Table 4) but were comparable to those of other studies not employing a database of predefined resistance mutations (14, 17, 35). The observed low sensitivities for some drugs were due to few strains belonging to the resistotype population included in the data set, the presence of additional resistance mutations in genes not assessed, or to unknown resistance mechanisms and not due to low coverages prohibiting the detection of mutations. The use of a curated SNP database containing high-confidence drug resistance mutations would improve sensitivity for some drugs where additional targets, less well associated with MIC increases, are known (34, 36). However, reliance on a predefined resistance mutation database comes at the cost of reduced sensitivity. After known phylogenetic mutations have been removed, it is important to treat any mutation in known target genes as potentially being involved in drug resistance. In cases where previously unknown mutations (i.e., not known to increase MICs and not a known phylogenetic SNP) in resistance-related genes are detected, genetic engineering and targeted DST are necessary to confirm or reject the drug resistance-conferring nature of a novel mutation to achieve high sensitivities and specificities for whole-genome sequencing-based DST.

Generating high-quality quantitative DST data using diverse M. tuberculosis strains is important to accurately define the ECOFF and subsequently guide treatment decisions. The two quantitative DST methods employed are difficult to standardize across laboratories, technically demanding, and at best challenging to scale up. Microtiter plate-based quantitative DST methods (37, 38) have the potential to aid in the generation of more high-quality DST data due to their standardized formulation and relative ease of application compared to established methods.

In conclusion, we demonstrate that MGIT 960 and 7H10 agar dilution-based phenotypic DST provide highly congruent classifications of strains into resistotype or wt populations. WGS has high predictive power to infer resistance profiles without the need for time-consuming phenotypic methods. Limitations due to overlapping distributions of wt and resistotype MICs, various MICs for the same mutations in different strains, presence of phylogenetic markers in resistance-associated genes, and rare resistance markers with low frequencies will likely be resolved by on-going large-scale projects (e.g., ReSeqTB and others [15, 39]), combining phenotypic DST with WGS of thousands of M. tuberculosis isolates. Our findings, together with those of on-going studies, will pave the way for the replacement of phenotypic DST with drug resistance profile prediction based on WGS in the coming years.

## MATERIALS AND METHODS

### M. tuberculosis isolates.

The initial data set consisted of 189 M. tuberculosis isolates. A subset of 61 strains was used to establish the phenotypic DST methodology. These 61 strains were collected by the Swiss National Center for Mycobacteria between 2004 and 2015 and represent a broad spectrum in geographic origin and drug resistance profiles (40–42). We then applied the quantitative DST methodology to 125 prospectively collected clinical isolates from clinics participating in the International Epidemiology Databases to Evaluate AIDS (IeDEA) (43) in Peru, Thailand, Ivory Coast, and the Democratic Republic of the Congo (see Table S3 in the supplemental material). Thirteen strains had to be excluded due to failed WGS (*n* = 4; failed library preparation due to low DNA quality), irreproducible DST results (*n* = 1), no growth in the 7H10 agar dilution assay (*n* = 3), duplication (*n* = 1), mixed cultures (*n* = 2; cross-contamination or patient infected with multiple strains), or transmission clusters (*n* = 2). The final set consisted of 176 strains.

### Phenotypic DST.

MGIT 960- and 7H10 agar dilution-based phenotypic DST were performed as described previously (40). Critical concentrations used for the classification of strains into resistant/susceptible aim to predict clinical outcome, i.e., treatment failure if a given strain is resistant at the critical concentration. However, critical concentrations should ideally be defined on the basis of the epidemiological cutoff (ECOFF; the highest wt MIC observed in the absence of any detectable resistance mechanism [23]), treatment outcomes, and pharmacokinetic and -dynamic data. As M. tuberculosis infections are treated with combination therapy, outcome data for single drugs are difficult to obtain (44). This calls for definition of critical concentrations solely based on the ECOFF (11). We therefore classified strains as belonging to the resistotype/wt populations on the basis of the detection of growth/no growth at the ECOFF derived from our data (45). Table 2 lists the ECOFFs used, Table S2 the drug concentrations tested with the MGIT 960 and 7H10 agar-dilution assays, and Table 3 the genes screened for mutations with WGS. Further details on how the phenotypic DST assays were performed are available in the supplemental material.

### Data analysis.

The categorical agreement between classification of strains into resistotype/wt populations using MGIT 960 and 7H10 agar dilution was based on detectable growth at the ECOFF (Table 2). The numerical variation between the two methods was quantified as the geometric standard deviation (SD; given with its standard error) of the ratio of MGIT 960 MIC to the agar dilution MIC, expressed as a number of 2-fold dilutions and denoted by σ. The geometric SD was computed by fitting a log-normal distribution to the MGIT 960 MIC/agar dilution MIC ratio as implemented in the R package fitdistrplus (v.1.0-9) (46). If the data were compatible with σ = 0, the geometric standard deviation could not be estimated and was defined as not applicable. The approach is a generalization of the Bland and Altman method (47), taking censoring of the data into account. Strains for which the MGIT 960 MIC and 7H10 agar dilution MIC were both left censored or both right censored were excluded, since no information on the ratio could be derived.

Goodman and Kruskal’s gamma was used to quantify the rank correlation between the two methods. No correlation could be calculated if the variance for either method was 0 and denoted not applicable.

Distributions of wt and mutant MICs were analyzed qualitatively based on the results of 7H10 agar dilution. We divided the data set into two groups: drugs for which the MIC distributions of wt and mutant strains did not overlap and those for which MIC distributions overlapped.

Sensitivities and specificities of WGS-based resistance profile inference were calculated based on the 7H10 agar dilution results for all drugs except pyrazinamide, for which the MGIT 960 results were used, based on growth/no growth at the ECOFF, derived from our data and the presence or absence of a putative resistance-associated mutation.

### Defining cutoffs for high- and low-level MICs.

The therapeutic window of a drug is defined as the concentration range within which a drug is considered to be effective and safe to use (19). Mutations can increase the MIC beyond the therapeutic window and render the drug clinically ineffective. Drugs may have large therapeutic windows beyond the ECOFF. For these, MIC increases caused by mutations may still be within the therapeutic window of a drug: these strains might still be treatable by increasing the drug dose. We analyzed the distribution of MICs of mutant strains and assessed if cutoffs for low-level (within the therapeutic window) and high-level (beyond the therapeutic window) MICs were definable. There were sufficient data available to define distinct cutoffs for low- and high-level MICs for isoniazid, rifampin, streptomycin, and amikacin. For mutations conferring resistance to other drugs assayed in this study, no distinct separation into resistotype populations with high- and low-level MICs was possible due to wide ranges of MICs conferred by the individual mutations or because the mutations conferred MICs beyond the therapeutic window.

### WGS and SNP calling.

WGS and data analysis were performed as previously described (48) and are summarized in the supplemental material. The performance of WGS-based DST greatly depends on the availability of robust markers of resistance. We therefore focused on a set of high-confidence resistance-associated genes (3, 14, 19) (Table 3). We additionally assessed the impact of *eis* promoter mutations on amikacin and capreomycin resistance, as the association of mutations in the *eis* promoter with resistance to the aforementioned drugs has been reported but is not well established (11, 49).

### Ethics.

Local institutional review board or ethics committee approval was obtained at all local study sites. Informed consent was obtained where requested per local regulations. This project was also approved by the Cantonal Ethics Committee in Bern, Switzerland.

## Supplementary Material

Supplemental file 1

Supplemental file 2
