# Supplementary material for: Whole-Genome Sequencing for Drug Resistance Profile Prediction in Mycobacterium tuberculosis
Source: Antimicrob Agents Chemother. 2019 Mar 27;63(4):e02175-18. doi: 10.1128/AAC.02175-18 (PMC6496161; doi:10.1128/AAC.02175-18)
Supplement: Supplemental file 1 [file AAC.02175-18-s0001.pdf]

Supplementary material

Supplementary text

Culture conditions

Strains were isolated from clinical material following established decontamination procedures. Bacteria were cultured in BD MGIT 960 tubes and Middlebrook 7H10 agar media. Strains collected as a part of the IeDEA study were cultured on Löwenstein-Jensen slants and sent to the National Center for Mycobacteria (University of Zürich, Zürich, Switzerland) for phenotypic drug susceptibility testing.

Phenotypic drug susceptibility testing – BD MGIT 960

DST for first- and second-line anti-TB drugs was performed using the Becton Dickinson (BD) MGIT 960 and EpiCenter devices equipped with the TB eXiST module (BD, Franklin Lakes, NJ, USA) (1). The MGIT 960 system was used for primary bacterial isolation and DST for first-line drugs (rifampin, isoniazid, ethambutol, pyrazinamide) as recommended by the manufacturer, using currently established epidemiological cut-offs (ECOFF – epidemiological cut-off, i.e the highest observed wild-type MIC (2)) summarized in Table 1 in the main text. MGIT tubes enriched with 0.8 ml of supplement (MGIT 960 OADC supplement; BD) were inoculated with 0.1 ml of the drug solution (supplementary Table 2) and 0.5 ml of the test strain suspension. For the drug-free growth control tube, the *M. tuberculosis* suspension was diluted 1:100 in sterile saline and 0.5 ml was inoculated into the tube (proportion testing).

Phenotypic drug susceptibility testing - Proportion method by 7H10 agar dilution

M. tuberculosis strains were grown in Middlebrook 7H9 liquid broth with 10% OADC supplement (BD) until McFarland 0.5 was reached. Using a replicator micropipettor robot VIAFLOW96 (Integra Biosciences, Zizers, Switzerland), 1 µl of the culture suspension was inoculated onto Middlebrook 7H10 agar plates with twofold serially diluted drug concentrations to determine the MIC (supplementary Table 2), whereby the MIC is defined as the lowest drug concentration that inhibits growth of more than 99% the bacteria during 21 days of incubation at 37°C (3). Agar plates were read automatically using an AID Microplate reader and automated software developed by AID (AID Diagnostika, Strassberg, Germany). 7H10 agar dilution MICs of the reference strains differed between the different

experimental runs by more than one dilution step for capreomycin, kanamycin A, amikacin and isoniazid. For these antibiotics, the MICs for all strains from the last run were rescaled by the differences observed for the reference strains. Phenotypic DST for all strains was performed at the Swiss National Center for Mycobacteria (University of Zürich, Zürich, Switzerland).

Whole genome sequencing

Libraries were prepared using the Illumina Nextera XT kit and sequenced on an Illumina HiSeq 2500, generating 125 bp paired-end reads. Sequencing was performed at the genomics facility of the ETHZ/University of Basel in Basel, Switzerland and at the Broad Institute, Cambridge, Massachusetts, United States.

The raw data was processed with an in-house python pipeline as follows: reads were adaptor clipped and quality trimmed with Trimmomatic (v.0.33), whereby resulting reads < 20 bp were discarded. Overlapping paired-end reads were merged using SeqPrep (https://github.com/jstjohn/SeqPrep). The processed reads were subsequentltly mapped to a reconstructed hypothetical MTBC ancestor (4) with BWA (v.0.7.12) (5). Duplicated reads were marked with Picard (v.2.1.1) (https://github.com/broadinstitute/picard) using the MarkDuplicates module. Local realignments of reads around indels were performed with the GATK (v.3.4.0) modules RealignerTargetCreator and IndelRealigner (6). Pileups were generated with Samtools (v.1.2) (7) and SNPs were subsequently called with VarScan (v.2.4.1) (8) using the following thresholds: minimum mapping & minimum base quality of 20, minimum read depth of 7X at a given position. For a SNP to be called, the alternative base call needed to be supported by at lest 5 reads without strand bias. Furthermore, SNPs were considered as fixed when they reached a frequency of ≥ 90%. The position was called as ancestral when the frequency was found to be < 10%. SNPs were annotated with SnpEff (v.4.11) (9) corresponding to the *M. tuberculosis* H37Rv reference annotation (NC_000962.3). SNPs in regions that share a minimum of 50 bp of sequence identity with other regions in the genome were excluded (10).

Variable SNP alignment and phylogenetic analysis

A variable SNP alignment was generated by concatenating all filtered SNPs in the dataset, whereby the IUPAC nucleotide ambiguity codes were used for unfixed positions (10% ≤ variant frequency < 90%). Positions were considered variable if at least one genome had

SNP-call at the position in question. If the SNP fell into an excluded region (see above) or was covered by less than 7 reads it was encoded as X in the alignment. If there was no sequence information at all available for a position, it was encoded as a gap. Furthermore, positions known to be involved in drug resistance were not considered in the alignment. The variable SNP alignment was used to infer a maximum likelihood phylogeny with RAxML (v.8.2.8) (11), using the general time-reversible model of sequence evolution and branch support values were inferred by bootstrapping the highest scoring maximum likelyhood tree (1000 pseudoreplicates). *Mycobacterium canettii* (SRR011186) was used to root the phylogeny. The genomes were classified into main and sub lineages based on the presence of previously established markers (12). Average genetic distances between strains based on fixed single nucleotide polymorphisms (SNP) were calculated with the ape package (v.4.1) (13) for R (v.3.3.3) (14). Two strains were defined as clustered if their average genetic distance was ≤ 12 SNPs (15) and one strain was subsequently omitted from the analysis at random. Phylogenetic trees with associated metadata were visualized with the R package ggtree (v.1.6.11) (16).

WGS-based resistance profile inferrence

The WGS data was screened for non-synonymous mutations and indels in genes known to be involved in drug resistance for the drugs assayed in *M. tuberculosis* (17, 18). Phylogenetic markers in drug resistance-associated genes (Table 2 – main text) shared by all strains belonging to a main- or sublineage (12, 19) (supplementary Table 1), as well as previously described phylogenetic markers (20), including main lineage markers derived from an unpublished collection of 400 phylogenetically diverse strains, were removed. After filtering for phylogenetic markers, all fixed non-synonymous polymorphisms in resistance-related genes were treated as linked to drug resistance. Where necessary, WGS results were complemented by molecular DST approaches as described previously (21).

Data availability

The datasets generated and analysed in the study were deposited at the National Center National Center for Biotechnology Information (NCBI) under the BioProject IDs PRJNA454477 - http://www.ncbi.nlm.nih.gov/bioproject/454477 and PRJNA300846 - https://www.ncbi.nlm.nih.gov/bioproject/PRJNA300846. Supplementary Table S3 provides a summary of sample identifyers and accession numbers and drug resistance information. The

quantitative readout of the 7H10 agar dilution-based DST and the results of the MGIT 960-based testing at the WHO-defined critical concentration are available upon request.

Supplementary Tables

Supplementary Table S1 List of major phylogenetic markers in drug-resistance related genes

Gene	Rv number	Position	Ref NT	Alt NT	Codon change	Lineage	Coll (12) sublineage	Comments
embC	Rv3793	4242182	G	T	Ala774Ser	L4	L4.3.3	Sublineage of L4.3.3
gid	Rv3919c	4407967	T	C	Leu79Ser	L4	L4.1	Sublineage of L4.1

Supplementary Table S2 List of drug concentrations tested for MGIT 960 and 7H10 agar dilution for the training and test set. ND = not determined

**Antibiotic**	**MGIT 960 concentrations (mg/L) Training set (n = 56)**	**MGIT 960 concentrations (mg/L) Test set (n = 120)**	**7H10 agar dilution concentration ranges (mg/L), log$_2$ steps, Training set (n = 56)**	**7H10 agar dilution concentration, ranges (mg/L), log$_2$ steps, Test set (n = 120)**
Ethionamide	1.25, 2.5, 5, 10, 25	ND	0.25-128	0.25-256
Ethambutol	1.25, 2.5, 5, 12.5, 50	5	0.5-64	0.5-64
Capreomycin	2.5, 5, 25	ND	0.5-64	0.5-64
Streptomycin	1, 4, 20	ND	0.06-128	0.06-128
Kanamycin A	1, 2, 4, 20	ND	0.06-128	0.06-128
Amikacin	1, 4, 20	1	0.06 - 128	0.06-128
Moxifloxacin	0.25, 0.5, 2.5, 7.5	0.25	0.016-32	0.016-32
Isoniazid	0.1, 1, 3, 10	0.1, 1	0.004-32	0.004-32
Rifampin	1, 4, 20	1	0.016-256	0.016-256
Rifabutin	0.1, 0.4, 2	ND	0.004-32	0.004-32
Pyrazinamide	100	100	ND	ND

Supplementary References

1. Springer B, Lucke K, Calligaris-Maibach R, Ritter C, Bottger EC. 2009. Quantitative Drug Susceptibility Testing of Mycobacterium tuberculosis by Use of MGIT 960 and EpiCenter Instrumentation. J Clin Microbiol 47:1773–1780.

2. Ängeby K, Juréen P, Kahlmeter G, Hoffner S, Schön T. 2012. Challenging a dogma: antimicrobial susceptibility testing breakpoints for Mycobacterium tuberculosis. Bull World Health Organ 90:693–698.

3. Sirgel FA, Wiid IJF, van Helden PD. 2009. Measuring Minimum Inhibitory Concentrations in Mycobacteria, p. 173–186. *In* Parish T., BA (ed.), Mycobacteria Protocols. Methods in Molecular Biology (Methods and Protocols). Humana Press, Totowa, NJ.

4. Comas I, Chakravartti J, Small PM, Galagan J, Niemann S, Kremer K, Ernst JD, Gagneux S. 2010. Human T cell epitopes of Mycobacterium tuberculosis are evolutionarily hyperconserved. Nat Genet 42:498–503.

5. Li H, Durbin R. 2009. Fast and accurate short read alignment with Burrows-Wheeler transform. Bioinformatics 25:1754–1760.

6. McKenna A, Hanna M, Banks E, Sivachenko A, Cibulskis K, Kernytsky A, Garimella K, Altshuler D, Gabriel S, Daly M, DePristo MA. 2010. The Genome Analysis Toolkit: A MapReduce framework for analyzing next-generation DNA sequencing data. Genome Res 20:1297–1303.

7. Li H, Handsaker B, Wysoker A, Fennell T, Ruan J, Homer N, Marth G, Abecasis G, Durbin R. 2009. The Sequence Alignment/Map format and SAMtools. Bioinformatics 25:2078–2079.

8. Koboldt DC, Zhang Q, Larson DE, Shen D, McLellan MD, Lin L, Miller C a, Mardis ER, Ding L, Wilson RK. 2012. VarScan 2: Somatic mutation and copy number alteration discovery in cancer by exome sequencing. Genome Res 22:568–576.

9. Cingolani P, Platts A, Wang LL, Coon M, Nguyen T, Wang L, Land SJ, Lu X, Ruden DM. 2012. A program for annotating and predicting the effects of single nucleotide polymorphisms, SnpEff. Fly (Austin) 6:80–92.

10. Stucki D. 2015. Transmission and evolution of Mycobacterium tuberculosis studied by whole genome sequencing and single nucleotide polymorphism-typing.

11. Stamatakis A. 2014. RAxML version 8: a tool for phylogenetic analysis and post-analysis of large phylogenies. Bioinformatics 30:1312–1313.

12. Coll F, McNerney R, Guerra-Assunção JA, Glynn JR, Perdigão J, Viveiros M, Portugal I, Pain A, Martin N, Clark TG. 2014. A robust SNP barcode for typing Mycobacterium tuberculosis complex strains. Nat Commun 5:4812.

13. Paradis E, Claude J, Strimmer K. 2004. APE: Analyses of Phylogenetics and Evolution in R language. Bioinformatics 20:289–290.

14. R Core Team. 2013. R: A Language and Environment for Statistical Computing. R Foundation for Statistical Computing, Vienna, Austria.

15. Walker TM, Ip CL, Harrell RH, Evans JT, Kapatai G, Dedicoat MJ, Eyre DW, Wilson DJ, Hawkey PM, Crook DW, Parkhill J, Harris D, Walker AS, Bowden R, Monk P, Smith EG, Peto TE. 2013. Whole-genome sequencing to delineate Mycobacterium tuberculosis outbreaks: a retrospective observational study. Lancet Infect Dis 13:137–146.

16. Yu G, Smith DK, Zhu H, Guan Y, Lam TT-Y. 2017. ggtree: an R package for visualization and annotation of phylogenetic trees with their covariates and other associated data. Methods Ecol Evol 8:28–36.

17. Gygli SM, Borrell S, Trauner A, Gagneux S. 2017. Antimicrobial resistance in Mycobacterium tuberculosis: mechanistic and evolutionary perspectives. FEMS Microbiol Rev 41:354–373.

18. Safi H, Lingaraju S, Amin A, Kim S, Jones M, Holmes M, McNeil M, Peterson SN, Chatterjee D, Fleischmann R, Alland D. 2013. Evolution of high-level ethambutol-resistant tuberculosis through interacting mutations in decaprenylphosphoryl-β-D-arabinose biosynthetic and utilization pathway genes. Nat Genet 45:1190–1197.

19. Stucki D, Brites D, Jeljeli L, Coscolla M, Liu Q, Trauner A, Fenner L, Rutaihwa L, Borrell S, Luo T, Gao Q, Kato-Maeda M, Ballif M, Egger M, Macedo R, Mardassi H, Moreno M, Tudo Vilanova G, Fyfe J, Globan M, Thomas J, Jamieson F, Guthrie JL,

Asante-Poku A, Yeboah-Manu D, Wampande E, Ssengooba W, Joloba M, Henry Boom W, Basu I, Bower J, Saraiva M, Vaconcellos SEG, Suffys P, Koch A, Wilkinson R, Gail-Bekker L, Malla B, Ley SD, Beck H-P, de Jong BC, Toit K, Sanchez-Padilla E, Bonnet M, Gil-Brusola A, Frank M, Penlap Beng VN, Eisenach K, Alani I, Wangui Ndung'u P, Revathi G, Gehre F, Akter S, Ntoumi F, Stewart-Isherwood L, Ntinginya NE, Rachow A, Hoelscher M, Cirillo DM, Skenders G, Hoffner S, Bakonyte D, Stakenas P, Diel R, Crudu V, Moldovan O, Al-Hajoj S, Otero L, Barletta F, Jane Carter E, Diero L, Supply P, Comas I, Niemann S, Gagneux S. 2016. Mycobacterium tuberculosis lineage 4 comprises globally distributed and geographically restricted sublineages. Nat Genet 48:1535–1543.

20. Coll F, McNerney R, Preston MD, Guerra-Assunção JA, Warry A, Hill-Cawthorne G, Mallard K, Nair M, Miranda A, Alves A, Perdigão J, Viveiros M, Portugal I, Hasan Z, Hasan R, Glynn JR, Martin N, Pain A, Clark TG. 2015. Rapid determination of anti-tuberculosis drug resistance from whole-genome sequences. Genome Med 7:51.

21. Ritter C, Lucke K, Sirgel FA, Warren RW, van Helden PD, Bottger EC, Bloemberg G V. 2014. Evaluation of the AID TB Resistance Line Probe Assay for Rapid Detection of Genetic Alterations Associated with Drug Resistance in Mycobacterium tuberculosis Strains. J Clin Microbiol 52:940–946.

Ethionamide (ETH)

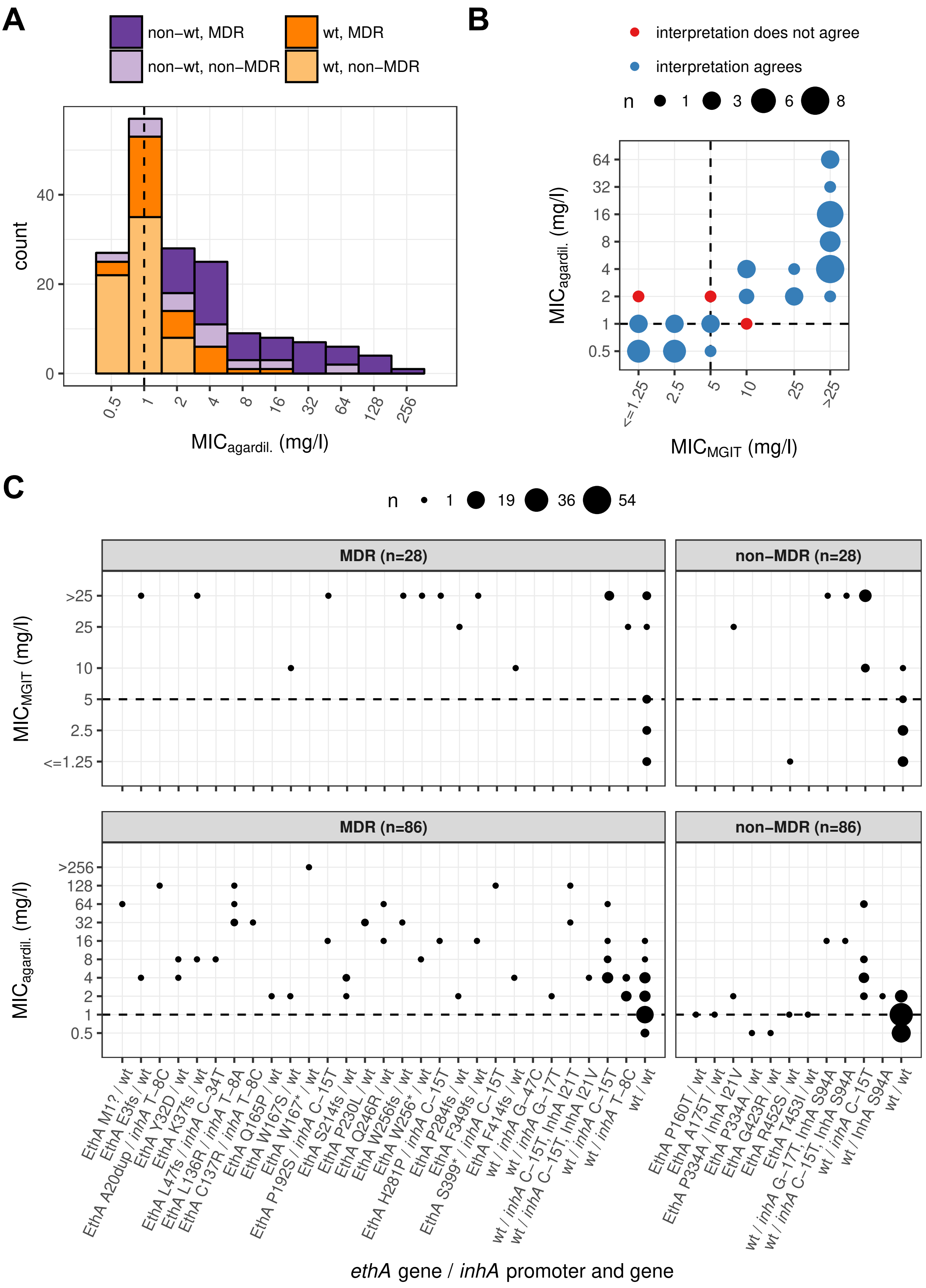

Supplementary Figure S1 - **A** Histogram of 7H10 agar dilution MICs for **ethionamide** resistance. **B** method agreement between phenotypic DST by MGIT 960 and 7H10 agar dilution. **C** MICs of *M. tuberculosis* strains harboring resistance mutations in target genes or for wt. Top panel MGIT 960, bottom panel 7H10 agar dilution. "M1?" indicates an initiator codon variant, "fs" indicates a frame shift variant, "*" indicates a stop-gain variant. In all panels dashed lines indicate the ECOFF (see Table 1 in the main text).

Ethambutol (EMB)

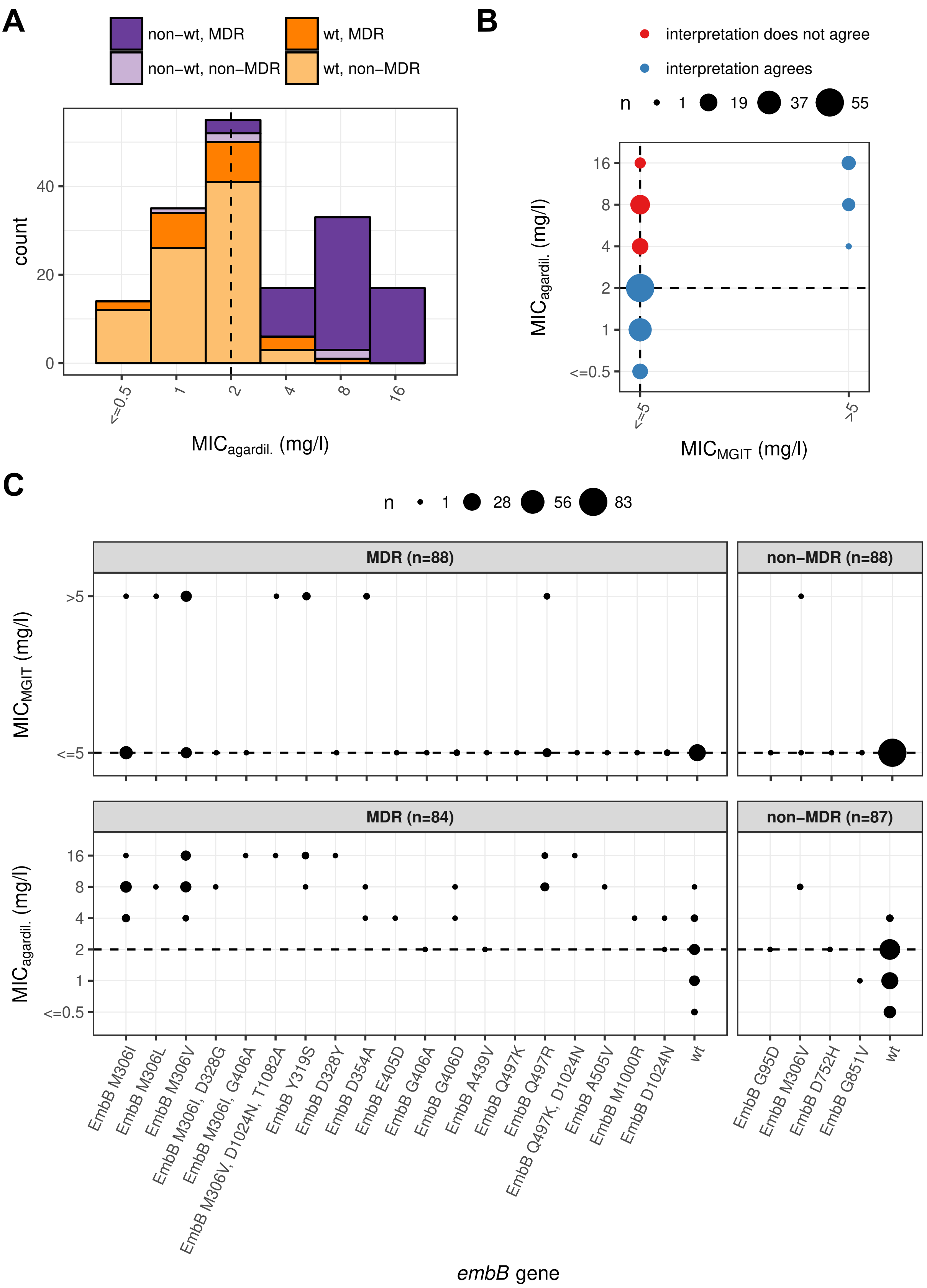

Supplementary Figure S2 - **A** Histogram of 7H10 agar dilution MICs for **ethambutol** resistance. **B** method agreement between phenotypic DST by MGIT 960 and 7H10 agar dilution. **C** MICs of *M. tuberculosis* strains harboring resistance mutations in target genes or for wt. Top panel MGIT 960, bottom panel 7H10 agar dilution. In all panels, dashed lines indicate the ECOFF (see Table 1 in the main text).

Streptomycin (STR)

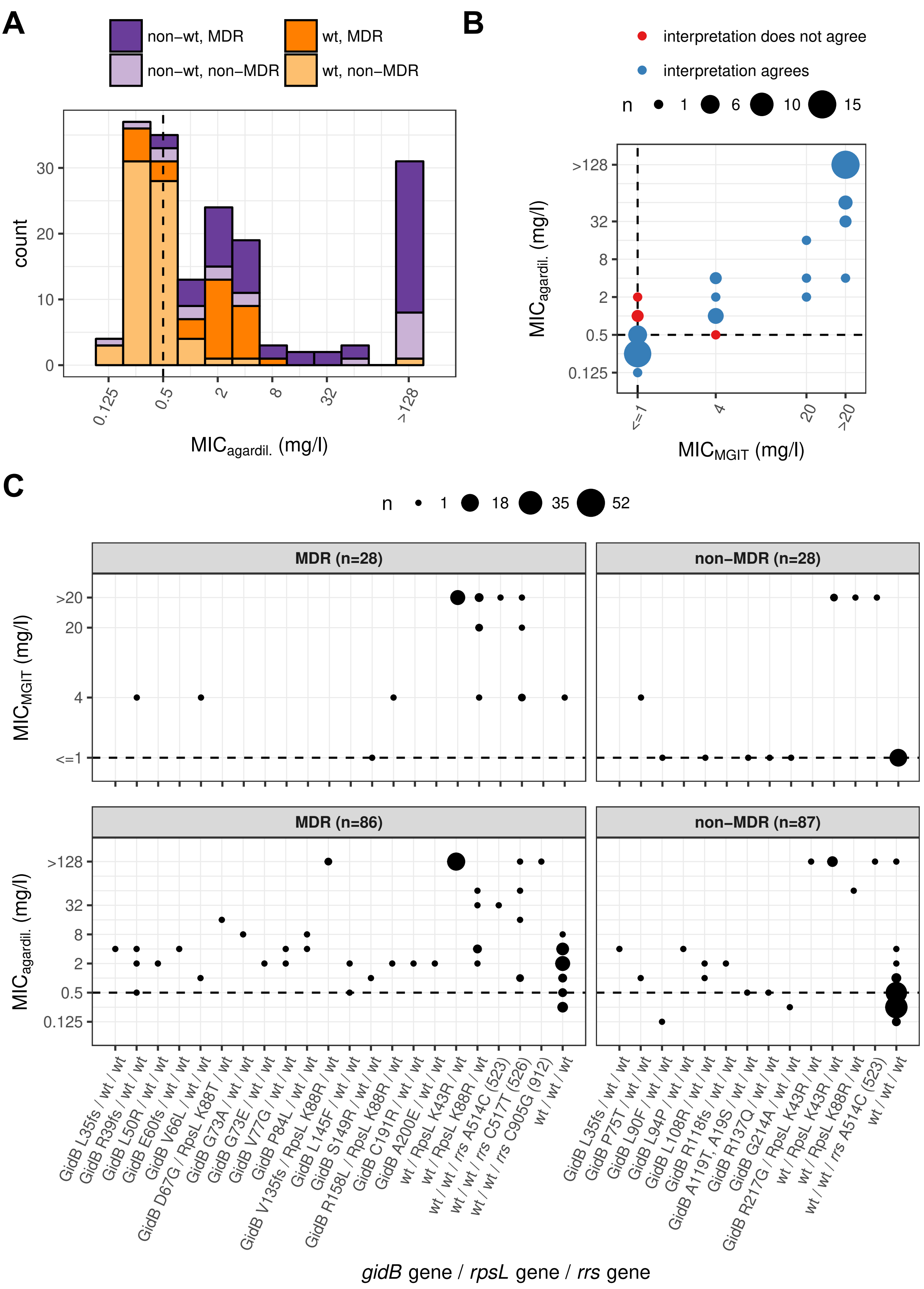

Supplementary Fgure S3 - A Histogram of 7H10 agar dilution MICs for **streptomycin** resistance. **B** method agreement between phenotypic DST by MGIT 960 and 7H10 agar dilution. **C** MICs of *M. tuberculosis* strains harboring resistance mutations in target genes or for wt. Numbers in parentheses indicate the corresponding *Escherichia coli* nucleotide numbering. Top panel MGIT 960, bottom panel 7H10 agar dilution. "fs" indicates a frame shift variant. In all panels, dashed lines indicate the ECOFF (see Table 1 in the main text).

Capreomycin (CAP)

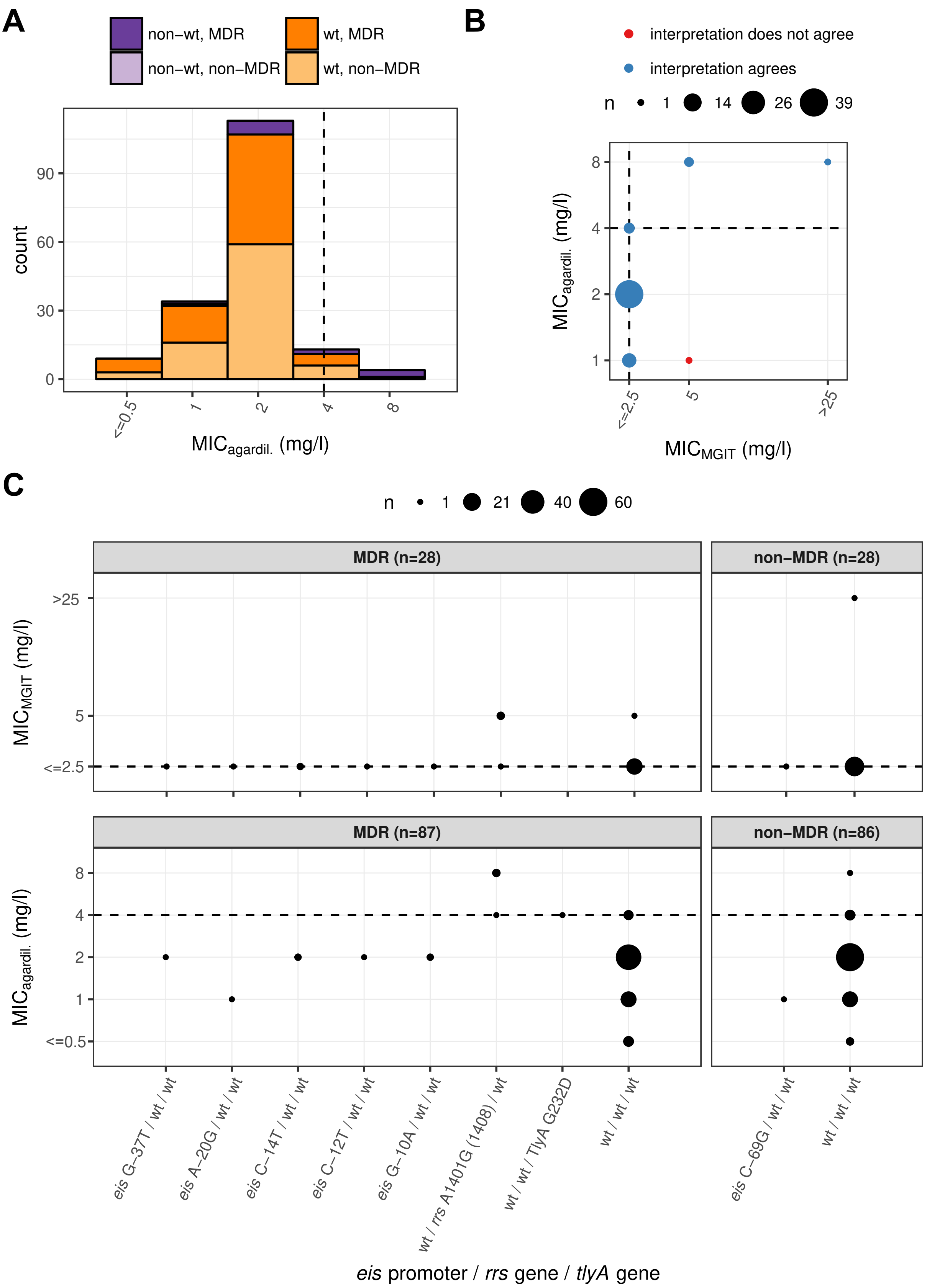

Supplementary Figure S4 - **A** Histogram of 7H10 agar dilution MICs for **capreomycin** resistance. **B** method agreement between phenotypic DST by MGIT 960 and 7H10 agar dilution. **C** MICs of *M. tuberculosis* strains harboring resistance mutations in target genes or for wt. Numbers in parentheses indicate the corresponding *Escherichia coli* nucleotide numbering. Top panel MGIT 960, bottom panel 7H10 agar dilution. In all panels, dashed lines indicate the ECOFF (see Table 1 in the main text).

Kanamycin A (KAN)

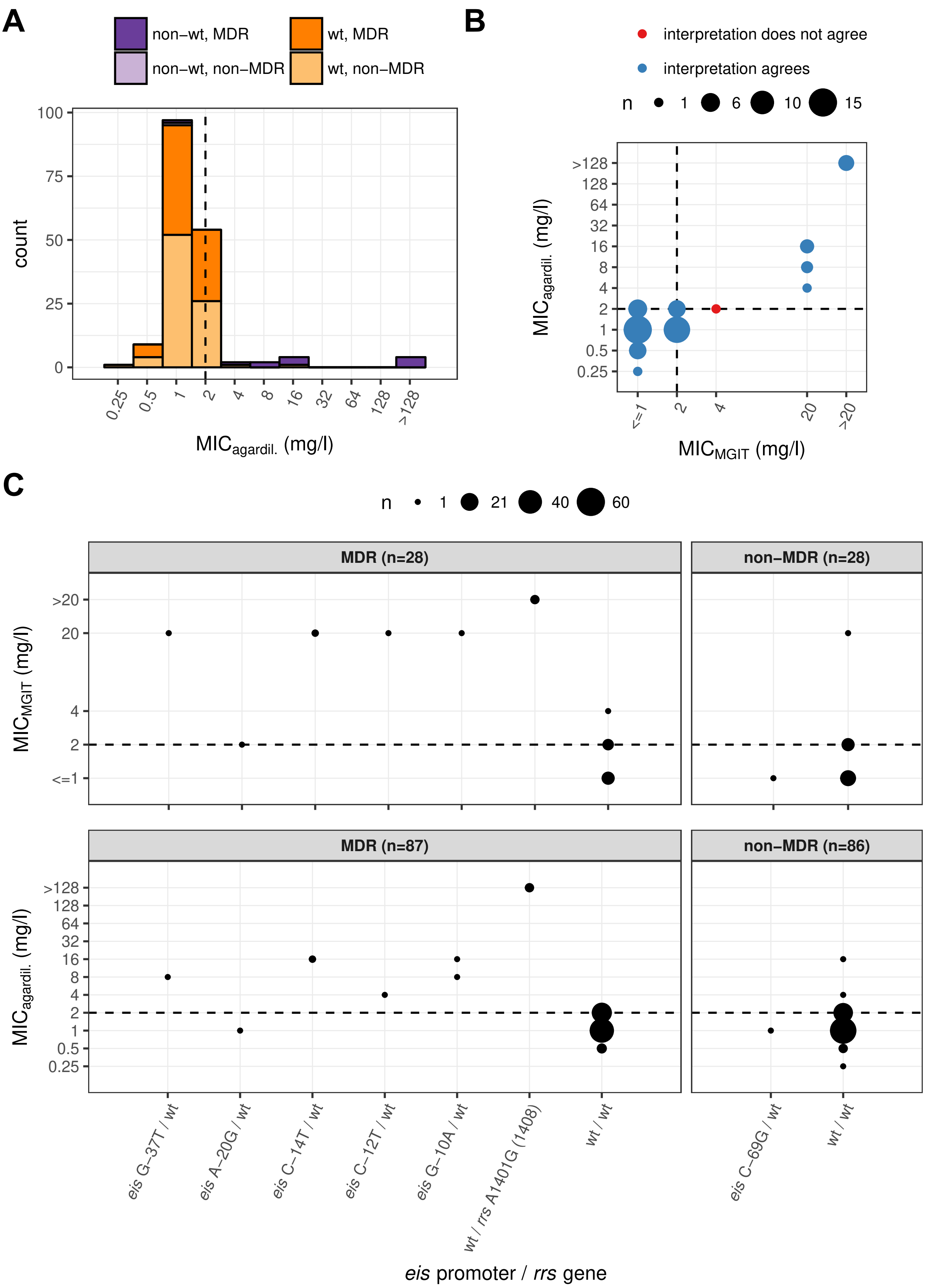

Supplementary Figure S5 - **A** Histogram of 7H10 agar dilution MICs for **kanamycin A** resistance. **B** method agreement between phenotypic DST by MGIT 960 and 7H10 agar dilution. **C** MICs of *M. tuberculosis* strains harboring resistance mutations in target genes or for wt. Numbers in parentheses indicate the corresponding *Escherichia coli* nucleotide numbering. Top panel MGIT 960, bottom panel 7H10 agar dilution. Numbers in parentheses indicate the corresponding *Escherichia coli* nucleotide number. In all panels, dashed lines indicate the ECOFF (see Table 1 in the main text).

Amikacin (AM)

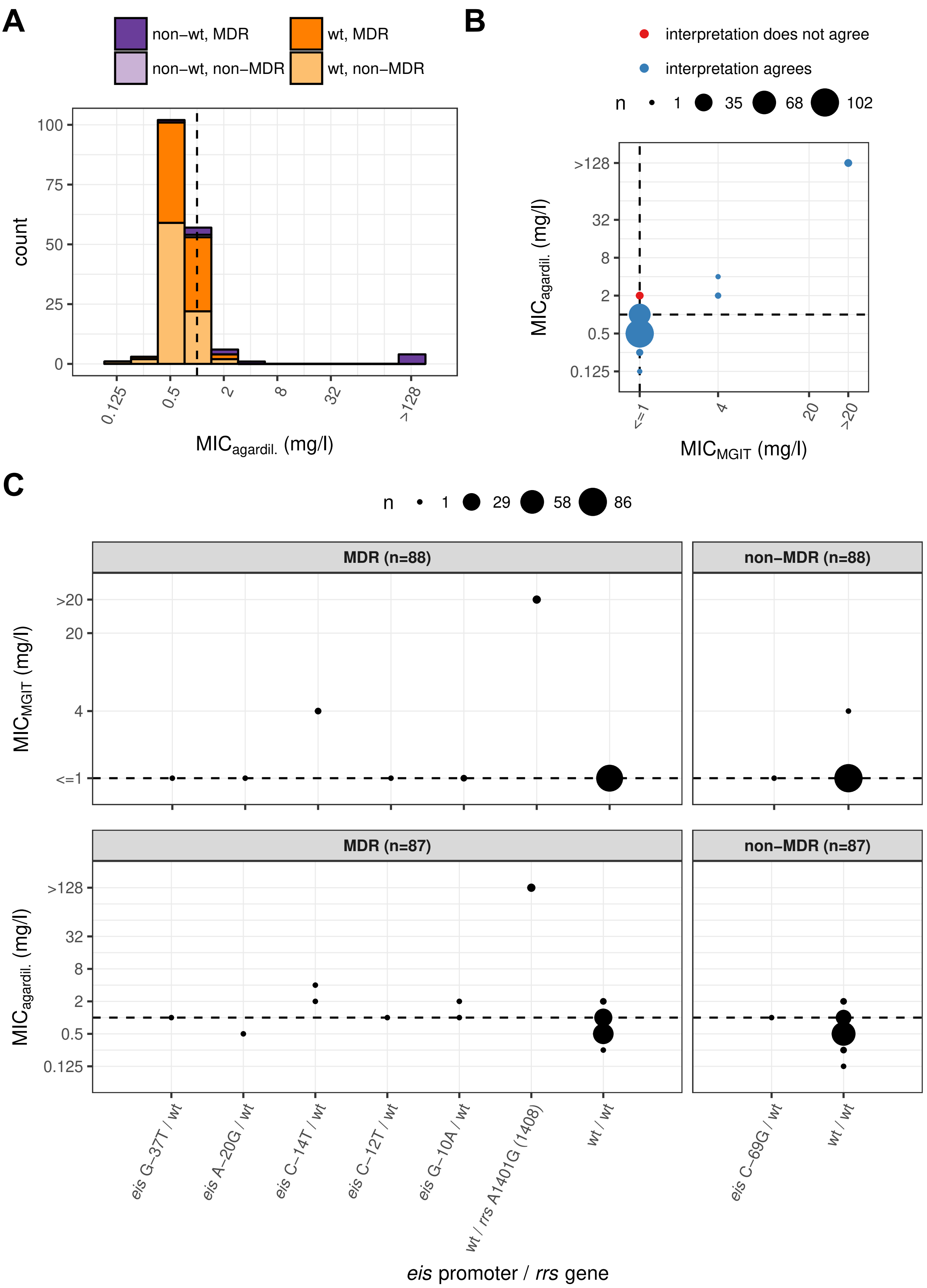

Supplementary Figure S6 - **A** Histogram of 7H10 agar dilution MICs for **amikacin** resistance. **B** method agreement between phenotypic DST by MGIT 960 and 7H10 agar dilution. **C** MICs of *M. tuberculosis* strains harboring resistance mutations in target genes or for wt. Numbers in parentheses indicate the corresponding *Escherichia coli* nucleotide numbering. Top panel MGIT 960, bottom panel 7H10 agar dilution. In all panels, dashed lines indicate the ECOFF (see Table 1 in the main text).

Moxifloxacin (MOX)

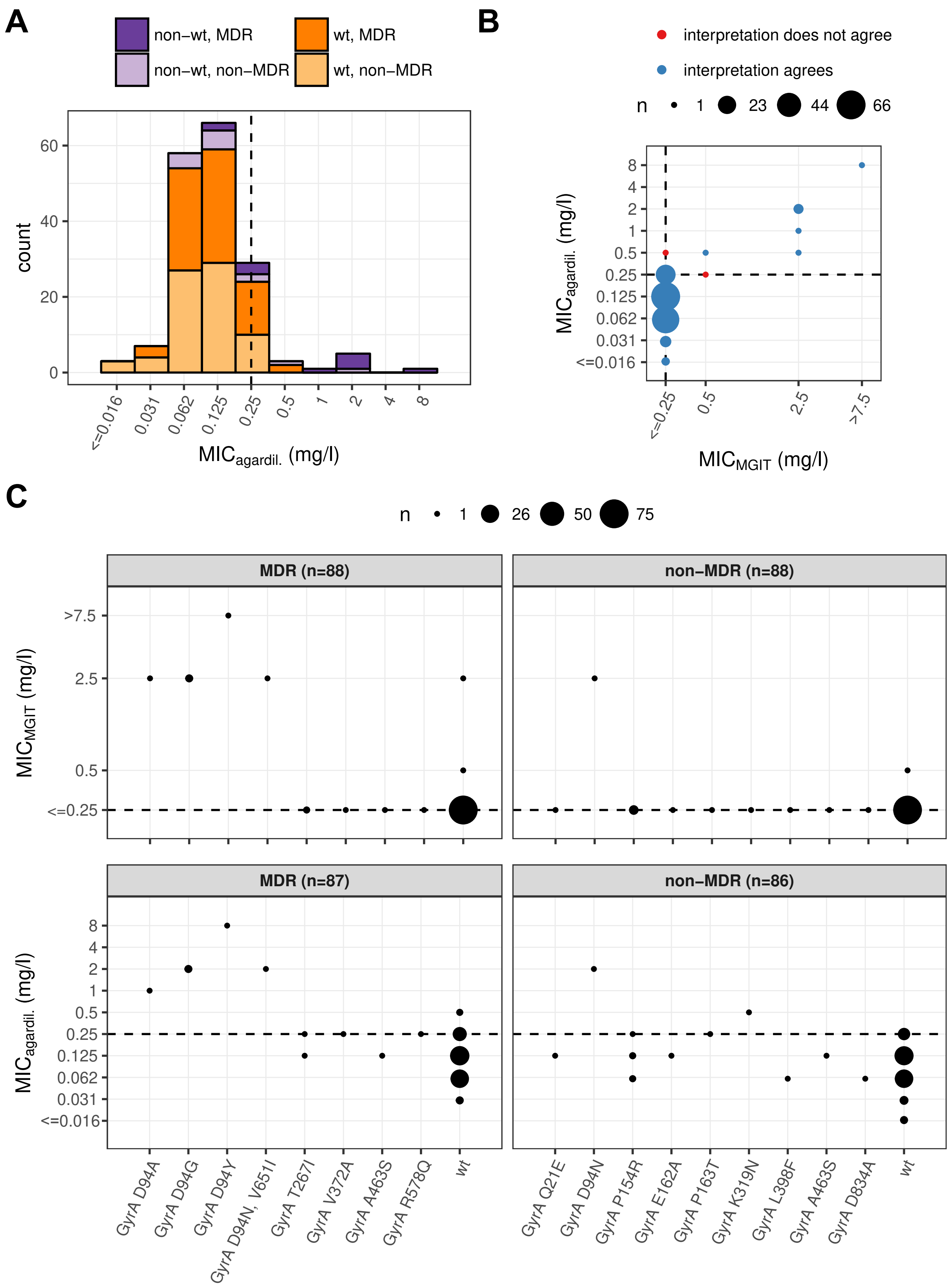

Supplementary Figure S7 - **A** Histogram of 7H10 agar dilution MICs for **moxifloxacin** resistance. **B** method agreement between phenotypic DST by MGIT 960 and 7H10 agar dilution. **C** MICs of *M. tuberculosis* strains harboring resistance mutations in target genes or for wt. Top panel MGIT 960, bottom panel 7H10 agar dilution. In all panels, dashed lines indicate the ECOFF (see Table 1 in the main text).

Isoniazid (INH)

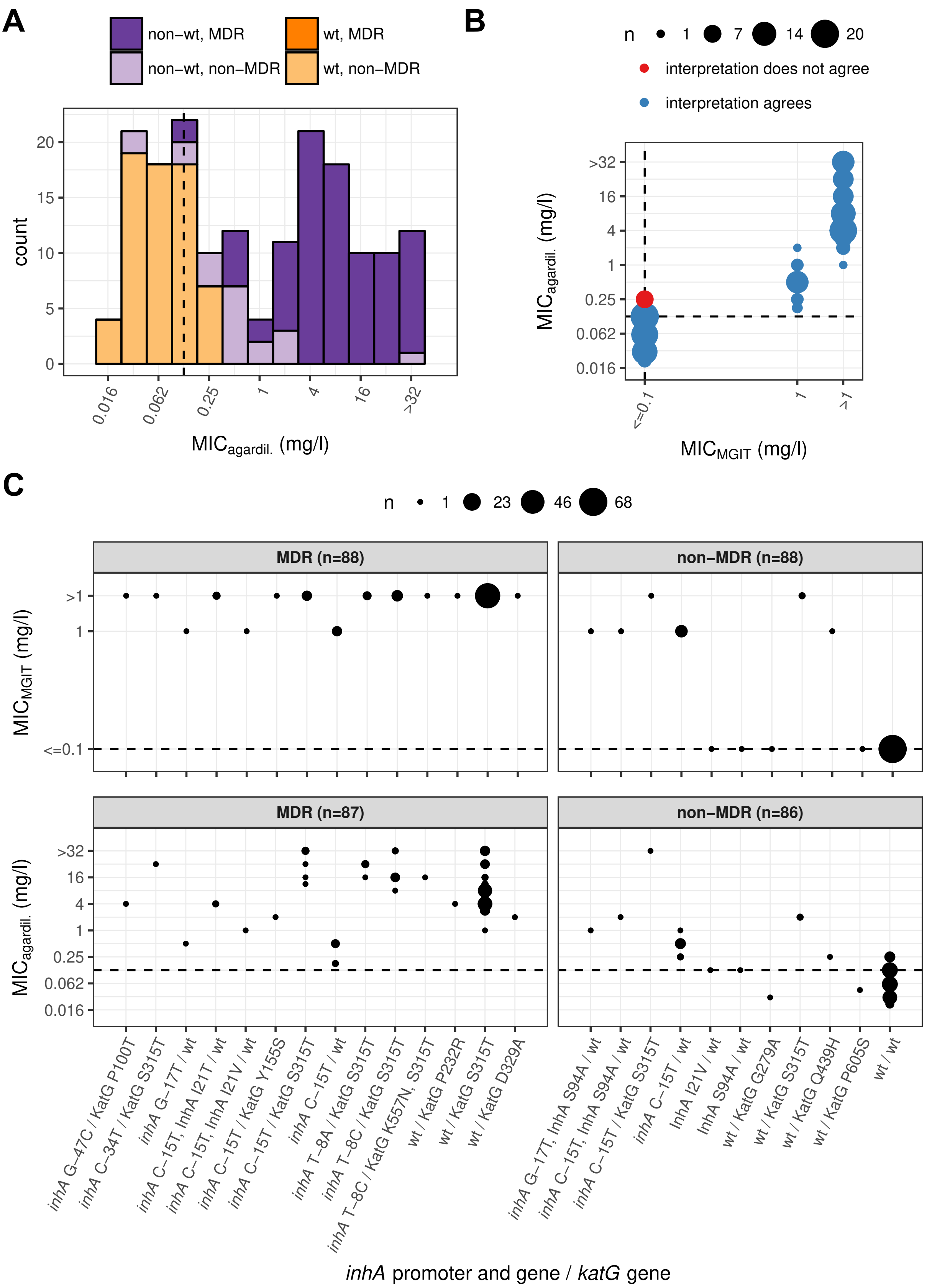

Supplementary Figure S8 - **A** Histogram of 7H10 agar dilution MICs for **isoniazid** resistance. **B** method agreement between phenotypic DST by MGIT 960 and 7H10 agar dilution. **C** MICs of *M. tuberculosis* strains harboring resistance mutations in target genes or for wt. Top panel MGIT 960, bottom panel 7H10 agar dilution. In all panels, dashed lines indicate the ECOFF (see Table 1 in the main text).

Rifampin (RMP)

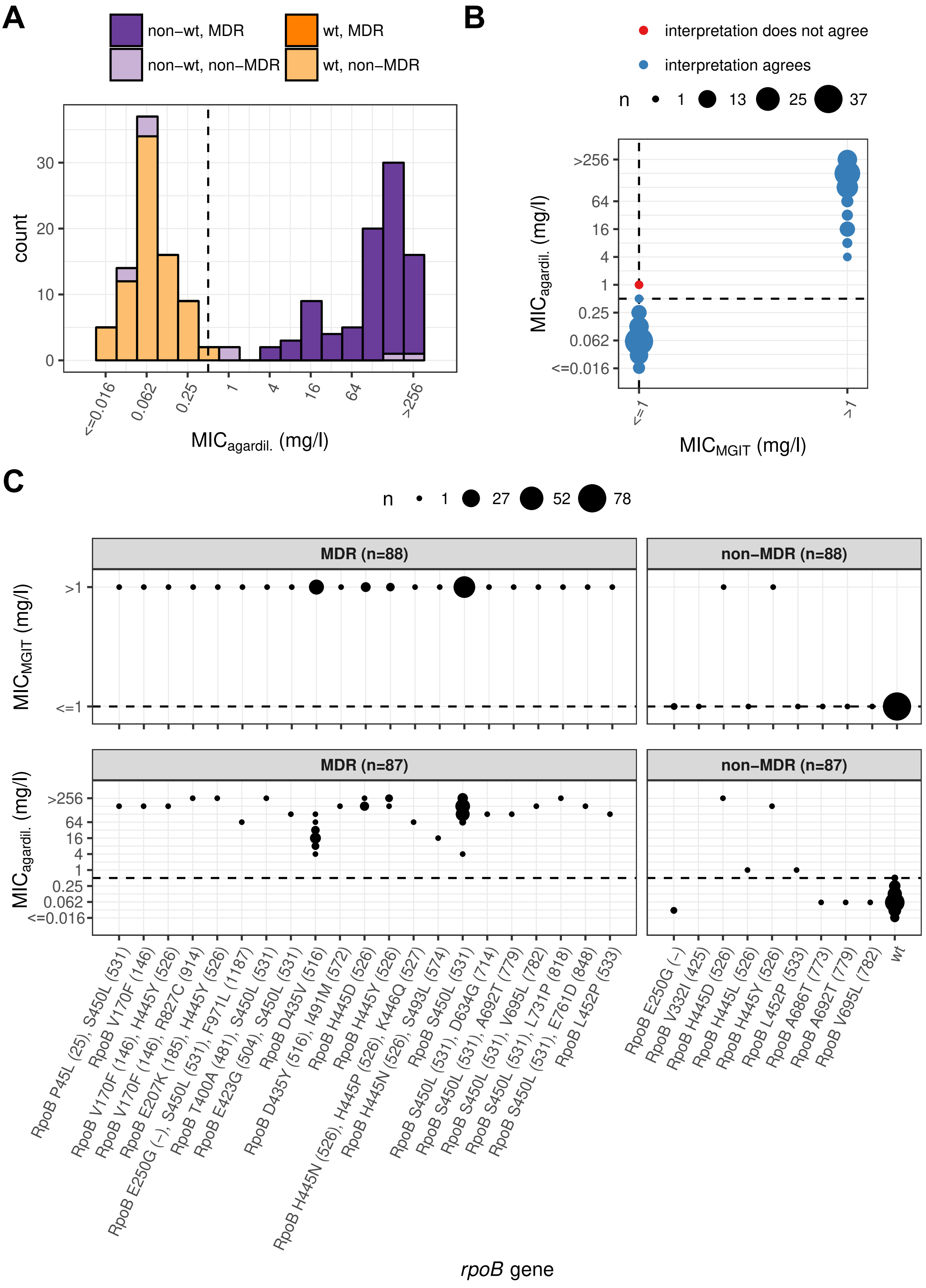

Supplementary Figure S9 - **A** Histogram of 7H10 agar dilution MICs for **rifampin** resistance. **B** method agreement between phenotypic DST by MGIT 960 and 7H10 agar dilution. **C** MICs of *M. tuberculosis* strains harboring resistance mutations in target genes or for wt. Top panel MGIT 960, bottom panel 7H10 agar dilution. Numbers in parentheses indicate the corresponding Escherichia coli codon numbers. In all panels, dashed lines indicate the ECOFF (see Table 1 in the main text).

Rifabutin (RBT)

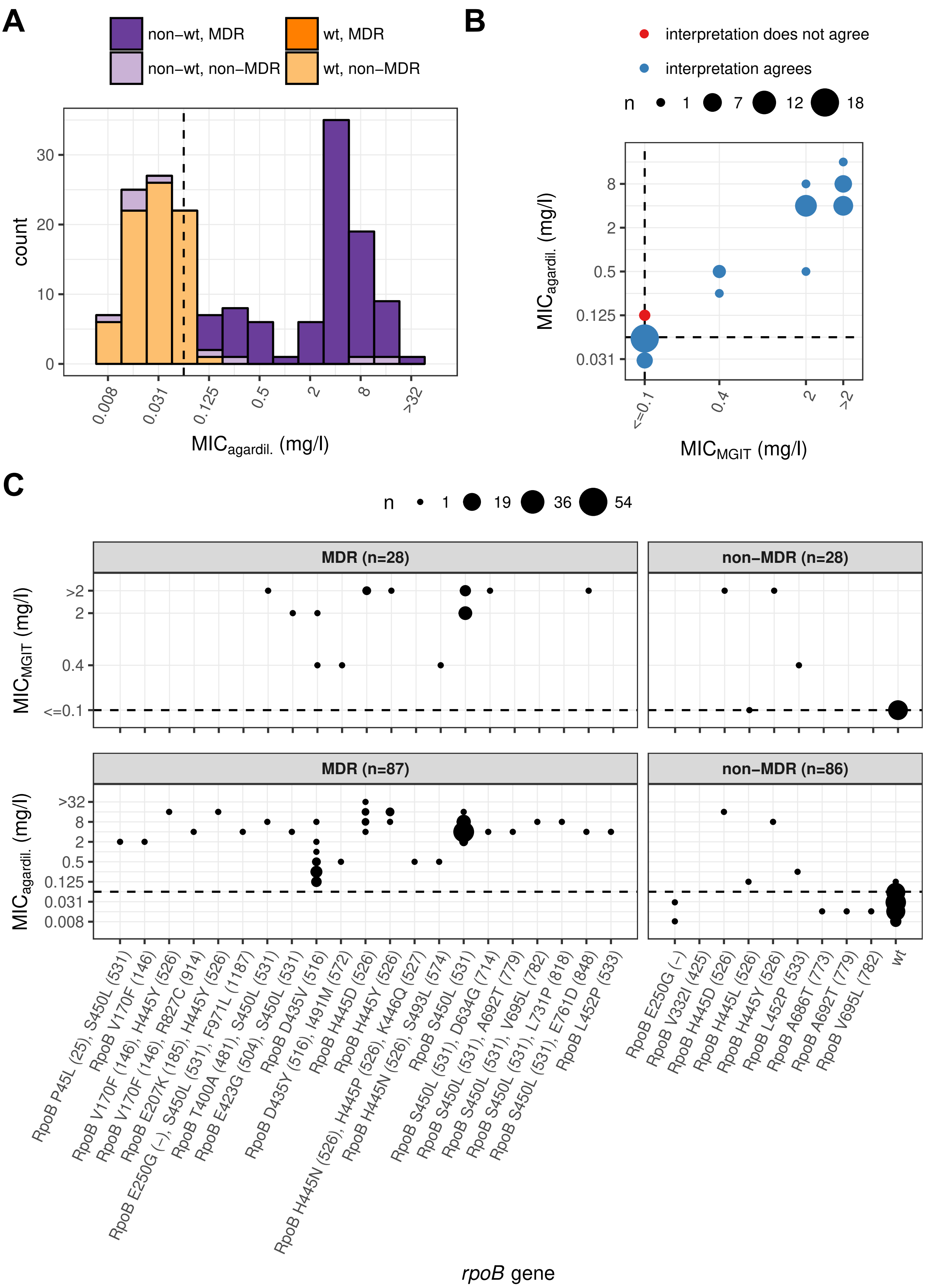

Supplementary Figure S10 - **A** Histogram of 7H10 agar dilution MICs for **rifabutin** resistance. **B** method agreement between phenotypic DST by MGIT 960 and 7H10 agar dilution. **C** MICs of *M. tuberculosis* strains harboring resistance mutations in target genes or for wt. Numbers in parentheses indicate the corresponding *Escherichia coli* codon numbers. Top panel MGIT 960, bottom panel 7H10 agar dilution. In all panels, dashed lines indicate the ECOFF (see Table 1 in the main text).

Pyrazinamide (PZA)

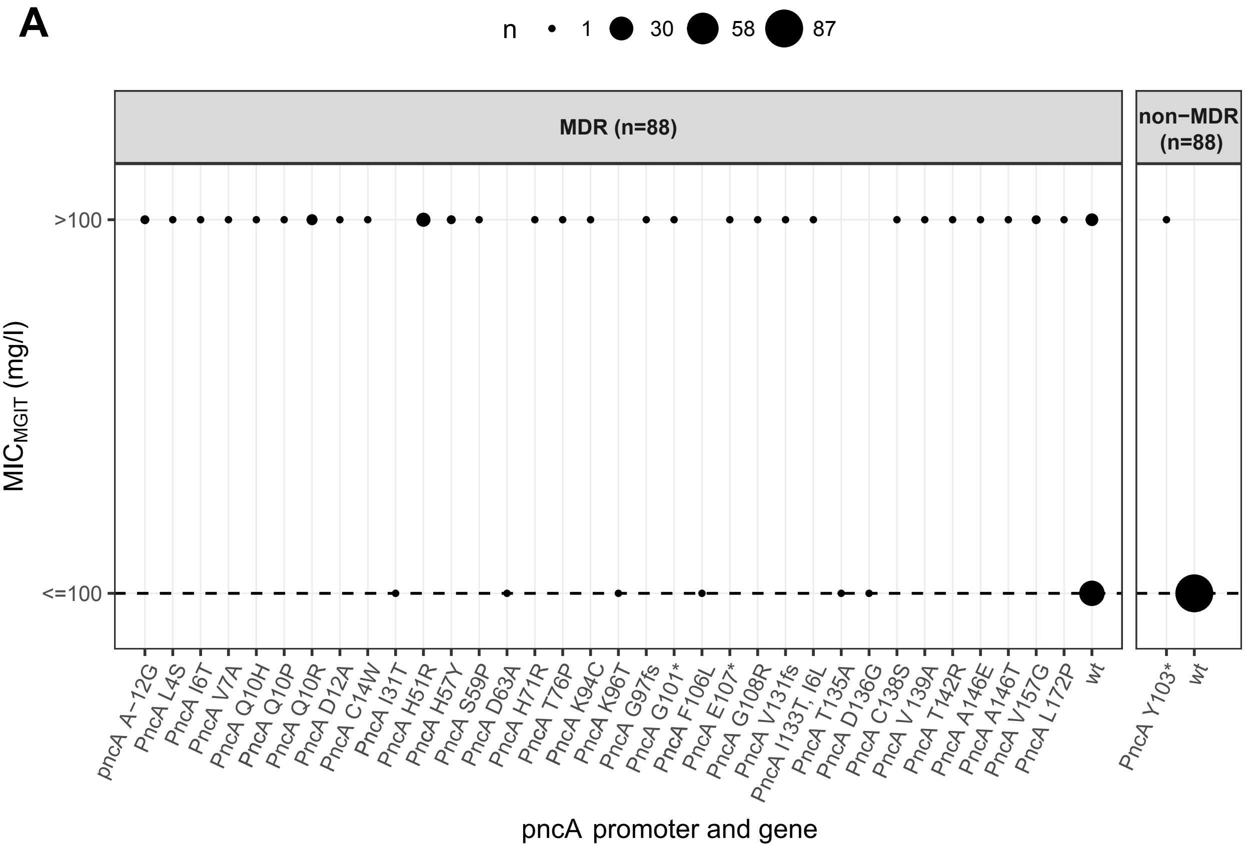

Supplementary Figure S11 - **A** MICs of M. tuberculosis strains harboring resistance mutations in the target gene for **pyrazinamide** resistance or for wt. Only results for MGIT 960 are available. "*" indicates a stop-gain variant. Dashed line indicates the ECOFF (see Table 1 in the main text).
